# Data from numerical analysis, simulation and experimental determinations performed to verify the sizing of an unbalanced capacitive compensator for three-phase four-wires networks

**DOI:** 10.1016/j.dib.2019.104366

**Published:** 2019-08-08

**Authors:** Adrian Pană, Alexandru Băloi, Florin Molnar-Matei

**Affiliations:** Politehnica University of Timisoara, Electrical Power Engineering Department, Romania

**Keywords:** Electrical power quality, Reactive power compensator, Balancing reactive compensator, Balancing capacitive compensator, Symmetrical component method

## Abstract

In a three-phase four-wire distribution network, the load unbalance can be compensated by the use of an unbalanced three-phase reactive compensator, containing six single-phase coils and capacitors, grouped into two distinct circuits, having Yn and delta connections. In (Pană et al., 2020) is presented a method of calculating the equivalent susceptances of the six reactive elements, so that they are capacitive or null. The paper demonstrates that a power factor improvement and a total or partial balancing of an unbalanced inductive load can be achieved by using an unbalanced capacitive compensator. The calculation method can be implemented in the control system of a SVC containing only single-phase capacitor banks. In this DiB article are presented additional data and information, obtained by numerical analysis, simulation-modeling and experimental determinations performed with the purpose of validation of the calculation method proposed in (Pană et al., 2020).

**Table:** Specifications Table

Subject area	Energy
More specific subject area	Electrical Distribution Networks, Electrical Power Quality
Type of data	Tables, graphs
How data was acquired	MathCAD Numerical analysis, Matlab-Simulink modeling an simulation, Experimental determinations using Mavowatt 230 - Power Quality Analizer, usual equipment from an electrical engineering laboratory
Data format	Raw and analyzed
Experimental factors	To verify the mathematical model's correctness, a three-phase circuit was built in the electrical engineering laboratory. The values of the circuit and of the supply voltage parameters were set as close as possible to the values considered in the numerical analysis.
Experimental features	The experiment was performed under real laboratory conditions, so that the effects on the data due to the non-symmetry and non-sinusoidal nature of the three-phase supply voltage set could not be avoided. The deviations coming from the difference between the real conditions of the experimental determinations and the ideal conditions, in which numerical analysis and software simulation have been performed, can be neglected.
Data source location	Politehnica University, Timisoara, Romania
Data accessibility	The data is within this article
Related research article	[1] Pană A., Băloi Al., Molnar-Matei F., New method for calculating the susceptances of a balancing capacitive compensator for a three-phase four-wire distribution network. International Journal of Electrical Power & Energy Systems 2020; 115:1–16, doi: 10.1016/j.ijepes.2019.105414

**Table:** 

**Value of the data** • The data are useful to demonstrate that an unbalanced three-phase active-inductive load can be fully or partially balanced, within a unitary power factor, through unbalanced capacitive compensation; • The data allow for a detailed verification of the energy mechanism of balancing an unbalanced active-inductive load through unbalanced capacitance compensation; • Data can be used to compare the various methods of sizing an unbalanced capacitive compensator; • The data are valuable in that it provides all the information necessary for the experimental verification of the correctness of the method and the behavior of the unbalanced capacitive compensator for a large number of particular cases of load; • The data may facilitate future research work on the design, construction and optimization of a *Static Var Compensator (SVC)* type *Adaptive Balancing Capacitive Compensator (ABCC)* in which control system an algorithm based on the method described in [1] has been implemented.

## 1 Data

This article presents the numeric data used in and obtained by:

- numerical analysis (MathCAD),
- modeling and simulation software (Matlab-Simulink),
- experimental determinations in the laboratory.

These were performed with the purpose of validating the correctness of the mathematical model of calculating (sizing) method of the susceptances of an unbalanced capacitive compensator designed to power factor improvement and load balancing in a three-phase four-wire network. The method was presented in [1] and consists in determining the computation relations for the compensator susceptances, valid for nine possible types of load. The load types were defined based on the values of the load sequence current components, components targeted by the compensator action.

In addition to the data and information provided in [1], this paper presents data from the numerical analysis of unbalanced capacitive compensation for 14 (13) particular cases of the load structure. To highlight the influence of the load power factor and the relative values of the negative and zero sequence components on the load balancing level that can be obtained by unbalanced capacitive compensation, all 14 (13) particular cases were chosen as belonging to type 1 of load, defined in [1]. The 14 (13) cases were divided into two groups as follows:

- the first group: the cases BCC1_1 … BCC1_7, where the unbalanced loads have the same values of the phase active powers but different values of the phase reactive powers, so that:

$$cos\varphi^{load}=0.447\ldots 0.909\text{,}\;k_{uI}^{-load}=0.42\ldots .0.51\text{,}\;k_{uI}^{0load}=0.179\ldots 0.037$$

-the second group: the cases BCC 1_8 … BCC 1_14, where the unbalanced loads have different structures for both phase active and reactive power, but the same power factor. However, the unbalance levels are different:

$$cos\varphi^{load}=0.447\;\;\;k_{uI}^{-load}=0.42\ldots .0.188\text{,}\;k_{uI}^{0load}=0.179\ldots 0.08$$

The input and output data of the numerical analysis for the first group of particular cases are presented in Table 1.1, Table 1.2, Table 1.3, Table 1.4, Table 1.5, and those for the second group are presented in Table 2.1, Table 2.2, Table 2.3, Table 2.4, Table 2.5. The parameter and electrical quantities notes are the same as in [1] and the output data of the analysis corresponds to the same three-phase circuit sections:

- Section 1 - unbalanced load;
- Section 2 - Yn compensator;
- Section 3 - Δ compensator;
- Section 4 - Yn+Δ compensator;
- Section 5 - PCC.

**Table 1.1:** Input data for numerical analysis of unbalanced capacitive compensation for the first group of particular load cases - Section 1 - Unbalanced load.

Comp.	Regime									
	Quantities			BCC 1_1	BCC 1_2	BCC 1_3	BCC 1_4	BCC 1_5	BCC 1_6	BCC 1_7
Load	1	$P_{A}^{load}$	[W]	200	200	200	200	200	200	200
	2	$P_{B}^{load}$	[W]	600	600	600	600	600	600	600
	3	$P_{C}^{load}$	[W]	400	400	400	400	400	400	400
	4	$P_{tot}^{load}$	[W]	1200	1200	1200	1200	1200	1200	1200
	5	$P_{av}^{load}$	[W]	400	400	400	400	400	400	400
	6	$R_{A}^{load}$	[Ω]	276.125	276.125	276.125	276.125	276.125	276.125	276.125
	7	$R_{B}^{load}$	[Ω]	92.041667	92.041667	92.041667	92.041667	92.041667	92.041667	92.041667
	8	$R_{C}^{load}$	[Ω]	138.0625	138.0625	138.0625	138.0625	138.0625	138.0625	138.0625
	9	$Q_{A}^{load}$	[var]	400	300	200	200	200	100	50
	10	$Q_{B}^{load}$	[var]	700	600	400	400	400	400	100
	11	$Q_{C}^{load}$	[var]	1300	1200	1100	800	600	500	400
	12	$Q_{tot}^{load}$	[var]	2400	2100	1700	1400	1200	1000	550
	13	$Q_{av}^{load}$	[var]	800	700	566.667	466.667	400	333.333	183.333
	14	$L_{A}^{load}$	[H]	0.439467	0.585955	0.878933	0.878933	0.878933	1.757866	3.515733
	15	$L_{B}^{load}$	[H]	0.251124	0.292978	0.439467	0.439467	0.439467	0.439467	1.757866
	16	$L_{C}^{load}$	[H]	0.13522	0.146489	0.159806	0.219733	0.292978	0.351573	0.439467
	17	$cos\varphi^{load}$	–	0.447214	0.496139	0.576683	0.650791	0.707107	0.768221	0.909065
	18	${\underline{I}}_{A}^{load}$	[A]	$1.903^{\underline{/-63.43{}^{\circ}}}$	$1.534^{\underline{/-56.31{}^{\circ}}}$	$1.204^{\underline{/-45{}^{\circ}}}$	$1.204^{\underline{/-45{}^{\circ}}}$	$1.204^{\underline{/-45{}^{\circ}}}$	$0.952^{\underline{/-26.57{}^{\circ}}}$	$0.877^{\underline{/-14.04{}^{\circ}}}$
	19	${\underline{I}}_{B}^{load}$	[A]	$3.923^{\underline{/-169.4{}^{\circ}}}$	$3.611^{\underline{/-165{}^{\circ}}}$	$3.069^{\underline{/-153.69{}^{\circ}}}$	$3.069^{\underline{/-153.69{}^{\circ}}}$	$3.069^{\underline{/-153.69{}^{\circ}}}$	$3.069^{\underline{/-153.69{}^{\circ}}}$	$2.588^{\underline{/-129.46{}^{\circ}}}$
	20	${\underline{I}}_{C}^{load}$	[A]	$5.788^{\underline{/47.1{}^{\circ}}}$	$5.383^{\underline{/48.43{}^{\circ}}}$	$4.981^{\underline{/49.98{}^{\circ}}}$	$3.806^{\underline{/56.57{}^{\circ}}}$	$3.069^{\underline{/63.69{}^{\circ}}}$	$2.725^{\underline{/68.66{}^{\circ}}}$	$2.407^{\underline{/75{}^{\circ}}}$
	21	${\underline{I}}_{load}^{+}$	[A]	$3.806^{\underline{/-63.43{}^{\circ}}}$	$3.431^{\underline{/-60.26{}^{\circ}}}$	$2.952^{\underline{/-54.78{}^{\circ}}}$	$2.615^{\underline{/-49.4{}^{\circ}}}$	$2.407^{\underline{/-45{}^{\circ}}}$	$2.216^{\underline{/-39.81{}^{\circ}}}$	$1.872^{\underline{/-24.62{}^{\circ}}}$
	22	${\underline{I}}_{load}^{-}$	[A]	$1.598^{\underline{/136.67{}^{\circ}}}$	$1.598^{\underline{/136.67{}^{\circ}}}$	$1.645^{\underline{/141.41{}^{\circ}}}$	$1.225^{\underline{/138.43{}^{\circ}}}$	$0.949^{\underline{/135{}^{\circ}}}$	$0.923^{\underline{/126.46{}^{\circ}}}$	$0.954^{\underline{/146.31{}^{\circ}}}$
	23	$k_{uI}^{-load}$	–	0.42	0.466	0.557	0.469	0.394	0.416	0.51
	24	${\underline{I}}_{load}^{0}$	[A]	$0.681^{\underline{/62.77{}^{\circ}}}$	$0.681^{\underline{/62.77{}^{\circ}}}$	$0.689^{\underline{/50.9{}^{\circ}}}$	$0.328^{\underline{/78.43{}^{\circ}}}$	$0.254^{\underline{/135{}^{\circ}}}$	$0.393^{\underline{/140.36{}^{\circ}}}$	$0.069^{\underline{/146.31{}^{\circ}}}$
	25	$k_{uI}^{0load}$	–	0.179	0.198	0.233	0.126	0.106	0.177	0.037
Weighting factors	26	$k_{Q}^{+}$	–	1	1	1	1	1	1	1
	27	$k_{Y}^{+}$	–	0.381198	0.381198	0.352271	0.246619	0.154701	0.203302	0.037235
	28	$k_{\Delta }^{+}$	–	0.618802	0.618802	0.647729	0.753381	0.845299	0.796698	0.962765
	29	$k^{-}$	–	1	0.937822	0.794678	0.761188	0.732051	0.574967	0.382149

**Table 1.2:** Output data for numerical analysis of unbalanced capacitive compensation for the first group of particular load cases - Section 2 - Yn compensator.

p.	Regime									
	Quantities			BCC 1_1	BCC 1_2	BCC 1_3	BCC 1_4	BCC 1_5	BCC 1_6	BCC 1_7
Compensator Yn	1	$C_{A}^{Y}$	[μF]	1.177463	0	0	0	0	0	0
	2	$C_{B}^{Y}$	[μF]	18.469082	16.216464	9.160849	8.774781	8.438896	9.942109	1.101328
	3	$C_{C}^{Y}$	[μF]	33.085678	29.924232	25.356766	11.125976	2.261195	1.1775987	0.079072
	4	$P_{A}^{Y}$	[W]	0	0	0	0	0	0	
	5	$P_{B}^{Y}$	[W]	0	0	0	0	0	0	
	6	$P_{C}^{Y}$	[W]	0	0	0	0	0	0	
	7	$Q_{A}^{Y}$	[var]	−20.428	0	0	0	0	0	−0
	8	$Q_{B}^{Y}$	[var]	−320.428	−281.347	−158.936	−152.238	−146.410	−172.490	−19.107
	9	$Q_{C}^{Y}$	[var]	−574.018	−519.169	−439.926	−193.03	−39.230	−30.812	−1.3719
	10	$Q_{tot}^{Y}$	[var]	−914.875	−800.515	−598.861	−345.267	−185.641	−203.302	−20.479
	11	$Q_{av}^{Y}$	[var]	−304.958	−266.838	−199.62	−115.089	−61.880	−67.768	−6.826
	12	${\underline{I}}_{A}^{Y}$	[A]	$0.087^{\underline{/90{}^{\circ}}}$	$0^{\underline{/0{}^{\circ}}}$	$0^{\underline{/0{}^{\circ}}}$	$0^{\underline{/0{}^{\circ}}}$	$0^{\underline{/0{}^{\circ}}}$	$0^{\underline{/0{}^{\circ}}}$	$0^{\underline{/0{}^{\circ}}}$
	13	${\underline{I}}_{B}^{Y}$	[A]	$1.364^{\underline{/-30{}^{\circ}}}$	$1.197^{\underline{/-30{}^{\circ}}}$	$0.676^{\underline{/-30{}^{\circ}}}$	$0.648^{\underline{/-30{}^{\circ}}}$	$0.623^{\underline{/-30{}^{\circ}}}$	$0.734^{\underline{/-30{}^{\circ}}}$	$0.081^{\underline{/-30{}^{\circ}}}$
	14	${\underline{I}}_{C}^{Y}$	[A]	$2.443^{\underline{/-150{}^{\circ}}}$	$2.209^{\underline{/-150{}^{\circ}}}$	$1.872^{\underline{/-150{}^{\circ}}}$	$0.821^{\underline{/-150{}^{\circ}}}$	$0.167^{\underline{/-150{}^{\circ}}}$	$0.131^{\underline{/-150{}^{\circ}}}$	$0.006^{\underline{/-150{}^{\circ}}}$
	15	${\underline{I}}_{Y}^{+}$	[A]	$1.298^{\underline{/90{}^{\circ}}}$	$1.135^{\underline{/90{}^{\circ}}}$	$0.849^{\underline{/90{}^{\circ}}}$	$0.49^{\underline{/90{}^{\circ}}}$	$0.263^{\underline{/90{}^{\circ}}}$	$0.288^{\underline{/90{}^{\circ}}}$	$0.029^{\underline{/90{}^{\circ}}}$
	16	${\underline{I}}_{Y}^{-}$	[A]	$0.681^{\underline{/-62.77{}^{\circ}}}$	$0.638^{\underline{/-62.77{}^{\circ}}}$	$0.547^{\underline{/-50.9{}^{\circ}}}$	$0.25^{\underline{/-78.43{}^{\circ}}}$	$0.186^{\underline{/-135{}^{\circ}}}$	$0.226^{\underline{/-140.36{}^{\circ}}}$	$0.026^{\underline{/-146.31{}^{\circ}}}$
	17	${\underline{I}}_{Y}^{0}$	[A]	$0.681^{\underline{/-117.23{}^{\circ}}}$	$0.638^{\underline{/-117.23{}^{\circ}}}$	$0.547^{\underline{/-129.1{}^{\circ}}}$	$0.25^{\underline{/-101.56{}^{\circ}}}$	$0.186^{\underline{/-45{}^{\circ}}}$	$0.226^{\underline{/-39.64{}^{\circ}}}$	$0.026^{\underline{/-33.69{}^{\circ}}}$

**Table 1.3:** Output data for numerical analysis of unbalanced capacitive compensation for the first group of particular load cases - Section 3 - Δ compensator.

Comp.	Regime									
	Quantities			BCC 1_1	BCC 1_2	BCC 1_3	BCC 1_4	BCC 1_5	BCC 1_6	BCC 1_7
Compensator Δ	1	$C_{AB}^{\Delta }$	[μF]	0.637129	0	0	0	0	0	0
	2	$C_{BC}^{\Delta }$	[μF]	13.948224	12.48344	10.578038	10.132244	9.744397	7.653439	5.086817
	3	$C_{CA}^{\Delta }$	[μF]	13.948224	12.48344	10.578038	10.132244	9.744397	7.653439	5.086817
	4	$P_{A}^{\Delta }$	[W]	200	187.564	158.936	152.238	146.410	114.993	76.43
	5	$P_{B}^{\Delta }$	[W]	−200	−187.564	−158.936	−152.238	−146.410	−114.993	−76.43
	6	$P_{C}^{\Delta }$	[W]	0	0	0	0	0	0	0
	7	$P_{tot}^{\Delta }$	[W]	0	0	0	−0	0	0	0
	8	$Q_{A}^{\Delta }$	[var]	−379.572	−324.871	−275.285	−263.683	−253.59	−199.174	−132.380
	9	$Q_{B}^{\Delta }$	[var]	−379.572	−324.871	−275.285	−263.683	−253.589	−199.174	−132.380
	10	$Q_{C}^{\Delta }$	[var]	−725.982	−649.742	−550.569	−527.366	−507.18	−398.349	−264.760
	11	$Q_{tot}^{\Delta }$	[var]	−1485.125	−1299.485	−1101.139	−1054.733	−1014.36	−796.698	−529.521
	12	$Q_{av}^{\Delta }$	[var]	−495.042	−433.162	−367.046	−351.578	−338.12	−265.566	−176.507
	13	${\underline{I}}_{A}^{\Delta }$	[A]	$1.826^{\underline{/62.21{}^{\circ}}}$	$1.596^{\underline{/60{}^{\circ}}}$	$1.353^{\underline{/60{}^{\circ}}}$	$1.296^{\underline{/60{}^{\circ}}}$	$1.246^{\underline{/60{}^{\circ}}}$	$0.979^{\underline{/60{}^{\circ}}}$	$0.65^{\underline{/60{}^{\circ}}}$
	14	${\underline{I}}_{B}^{\Delta }$	[A]	$1.826^{\underline{/-2.21{}^{\circ}}}$	$1.596^{\underline{/0{}^{\circ}}}$	$1.353^{\underline{/0{}^{\circ}}}$	$1.296^{\underline{/0{}^{\circ}}}$	$1.246^{\underline{/0{}^{\circ}}}$	$0.979^{\underline{/0{}^{\circ}}}$	$0.65^{\underline{/0{}^{\circ}}}$
	15	${\underline{I}}_{C}^{\Delta }$	[A]	$3.089^{\underline{/-150{}^{\circ}}}$	$2.765^{\underline{/-150{}^{\circ}}}$	$2.343^{\underline{/-150{}^{\circ}}}$	$2.244^{\underline{/-150{}^{\circ}}}$	$2.158^{\underline{/-150{}^{\circ}}}$	$1.695^{\underline{/-150{}^{\circ}}}$	$1.127^{\underline{/-150{}^{\circ}}}$
	16	${\underline{I}}_{\Delta }^{+}$	[A]	$2.107^{\underline{/90{}^{\circ}}}$	$1.843^{\underline{/90{}^{\circ}}}$	$1.562^{\underline{/90{}^{\circ}}}$	$1.496^{\underline{/90{}^{\circ}}}$	$1.439^{\underline{/90{}^{\circ}}}$	$1.13^{\underline{/90{}^{\circ}}}$	$0.751^{\underline{/90{}^{\circ}}}$
	17	${\underline{I}}_{\Delta }^{-}$	[A]	$0.983^{\underline{/-30{}^{\circ}}}$	$0.922^{\underline{/-30{}^{\circ}}}$	$0.781^{\underline{/-30{}^{\circ}}}$	$0.748^{\underline{/-30{}^{\circ}}}$	$0.719^{\underline{/-30{}^{\circ}}}$	$0.565^{\underline{/-30{}^{\circ}}}$	$0.376^{\underline{/-30{}^{\circ}}}$
	18	${\underline{I}}_{\Delta }^{0}$	[A]	$0^{\underline{/0{}^{\circ}}}$	$0^{\underline{/0{}^{\circ}}}$	$0^{\underline{/0{}^{\circ}}}$	$0^{\underline{/0{}^{\circ}}}$	$0^{\underline{/0{}^{\circ}}}$	$0^{\underline{/0{}^{\circ}}}$	$0^{\underline{/0{}^{\circ}}}$

**Table 1.4:** Output data for numerical analysis of unbalanced capacitive compensation for the first group of particular load cases - Section 4 - Yn+Δ compensator.

Comp.	Regime									
	Quantities			BCC 1_1	BCC 1_2	BCC 1_3	BCC 1_4	BCC 1_5	BCC 1_6	BCC 1_7
Compensator Yn + Δ	1	$P_{A}^{comp}$	[W]	200	187.564	158.936	152.238	146.41	114.993	76.43
	2	$P_{B}^{comp}$	[W]	−200	−187.564	−158.936	−152.238	−146.41	−114.993	−76.43
	3	$P_{C}^{comp}$		0	0	0	0	0	0	0
	4	$P_{tot}^{comp}$		−0	−0	−0	−0	0	0	0
	5	$P_{av}^{comp}$	[W]	−0	−0	−0	−0	0	0	0
	6	$Q_{A}^{comp}$	[var]	−400	−324.871	−275.285	−263.683	−253.59	−199.174	−132.38
	7	$Q_{B}^{comp}$	[var]	−700	−606.218	−434.22	−415.921	−400	−371.664	−151.488
	8	$Q_{C}^{comp}$	[var]	−1300	−1168.911	−990.495	−720.396	−546.41	−429.161	−266.132
	9	$Q_{tot}^{comp}$	[var]	−2400	−2100	−1700	−1400	−1200	−1000	−550
	10	$Q_{av}^{comp}$	[var]	−800	−700	−566.667	−466.667	−400	−333.333	−183.333
	11	${\underline{I}}_{A}^{comp}$	[A]	$1.903^{\underline{/63.43{}^{\circ}}}$	$1.596^{\underline{/60{}^{\circ}}}$	$1.353^{\underline{/60{}^{\circ}}}$	$1.296^{\underline{/60{}^{\circ}}}$	$1.246^{\underline{/60{}^{\circ}}}$	$0.979^{\underline{/60{}^{\circ}}}$	$0.65^{\underline{/60{}^{\circ}}}$
	12	${\underline{I}}_{B}^{comp}$	[A]	$3.098^{\underline{/-14.05{}^{\circ}}}$	$2.7^{\underline{/-12.81{}^{\circ}}}$	$1.968^{\underline{/-9.9{}^{\circ}}}$	$1.885^{\underline{/-9.9{}^{\circ}}}$	$1.813^{\underline{/-9.9{}^{\circ}}}$	$1.656^{\underline{/-12.81{}^{\circ}}}$	$0.722^{\underline{/-3.23{}^{\circ}}}$
	13	${\underline{I}}_{C}^{comp}$	[A]	$5.532^{\underline{/-150{}^{\circ}}}$	$4.974^{\underline{/-150{}^{\circ}}}$	$4.215^{\underline{/-150{}^{\circ}}}$	$3.066^{\underline{/-150{}^{\circ}}}$	$2.325^{\underline{/-150{}^{\circ}}}$	$1.826^{\underline{/-150{}^{\circ}}}$	$1.132^{\underline{/-150}}$
	14	${\underline{I}}_{comp}^{+}$	[A]	$3.404^{\underline{/90{}^{\circ}}}$	$2.979^{\underline{/90{}^{\circ}}}$	$2.411^{\underline{/90{}^{\circ}}}$	$1.986^{\underline{/90{}^{\circ}}}$	$1.702^{\underline{/90{}^{\circ}}}$	$1.418^{\underline{/90{}^{\circ}}}$	$0.78^{\underline{/90{}^{\circ}}}$
	15	${\underline{I}}_{comp}^{-}$	[A]	$1.598^{\underline{/-43.33{}^{\circ}}}$	$1.499^{\underline{/-43.33{}^{\circ}}}$	$1.307^{\underline{/-38.59{}^{\circ}}}$	$0.933^{\underline{/-41.57{}^{\circ}}}$	$0.695^{\underline{/-45{}^{\circ}}}$	$0.531^{\underline{/-53.54{}^{\circ}}}$	$0.365^{\underline{/-33.69{}^{\circ}}}$
	16	${\underline{I}}_{comp}^{0}$	[A]	$0.681^{\underline{/-117.23{}^{\circ}}}$	$0.638^{\underline{/-117.23{}^{\circ}}}$	$0.547^{\underline{/-129.1{}^{\circ}}}$	$0.25^{\underline{/-101.57{}^{\circ}}}$	$0.186^{\underline{/-45{}^{\circ}}}$	$0.226^{\underline{/-39.64{}^{\circ}}}$	$0.026^{\underline{/-33.69{}^{\circ}}}$

**Table 1.5:** Output data for numerical analysis of unbalanced capacitive compensation for the first group of particular load cases - Section 5 - PCC.

Comp.	Regime									
	Quantities			BCC 1_1	BCC 1_2	BCC 1_3	BCC 1_4	BCC 1_5	BCC 1_6	BCC 1_7
PCC	1	$P_{A}^{PCC}$	[W]	400	387.564	358.936	352.238	346.410	314.993	276.429732
	2	$P_{B}^{PCC}$	[W]	400	412.436	441.064	447.762	453.590	485.007	523.570268
	3	$P_{C}^{PCC}$	[W]	400	400	400	400	400	400	400
	4	$P_{tot}^{PCC}$	[W]	1200	1200	1200	1200	1200	1200	1200
	5	$P_{av}^{PCC}$	[W]	400	400	400	400	400	400	400
	6	$Q_{A}^{PCC}$	[var]	0	−24.871	−75.285	−63.683	−53.590	−99.174	−82.380
	7	$Q_{B}^{PCC}$	[var]	0	−6.218	−34.220	−15.921	0	28.335	−51.488
	8	$Q_{C}^{PCC}$	[var]	0	31.089	109.505	79.604	53.590	70.839	133.868
	9	$Q_{tot}^{PCC}$	[var]	0	0	0	0	0	0	0
	10	$Q_{av}^{PCC}$	[var]	0	0	0	0	0	0	0
	11	$cos\varphi^{PCC}$	–	1	1	1	1	1	1	1
	12	${\underline{I}}_{A}^{PCC}$	[A]	$1.702^{\underline{/0{}^{\circ}}}$	$1.653^{\underline{/3.67{}^{\circ}}}$	$1.561^{\underline{/11.85{}^{\circ}}}$	$1.523^{\underline{/10.25{}^{\circ}}}$	$1.492^{\underline{/8.79{}^{\circ}}}$	$1.405^{\underline{/17.48{}^{\circ}}}$	$1.227^{\underline{/16.59{}^{\circ}}}$
	13	${\underline{I}}_{B}^{PCC}$	[A]	$1.702^{\underline{/-120{}^{\circ}}}$	$1.755^{\underline{/-119.14{}^{\circ}}}$	$1.883^{\underline{/-115.56{}^{\circ}}}$	$1.907^{\underline{/-117.96{}^{\circ}}}$	$1.930^{\underline{/-120{}^{\circ}}}$	$2.067^{\underline{/-123.34{}^{\circ}}}$	$2.239^{\underline{/-114.38{}^{\circ}}}$
	14	${\underline{I}}_{C}^{PCC}$	[A]	$1.702^{\underline{/120{}^{\circ}}}$	$1.707^{\underline{/115.56{}^{\circ}}}$	$1.765^{\underline{/104.69{}^{\circ}}}$	$1.736^{\underline{/108.74{}^{\circ}}}$	$1.717^{\underline{/112.37{}^{\circ}}}$	$1.729^{\underline{/109.96{}^{\circ}}}$	$1.795^{\underline{/101.5{}^{\circ}}}$
	15	${\underline{I}}_{PCC}^{+}$	[A]	$1.702^{\underline{/0{}^{\circ}}}$	$1.702^{\underline{/0{}^{\circ}}}$	$1.702^{\underline{/0{}^{\circ}}}$	$1.702^{\underline{/0{}^{\circ}}}$	$1.702^{\underline{/0{}^{\circ}}}$	$1.702^{\underline{/0{}^{\circ}}}$	$1.702^{\underline{/0{}^{\circ}}}$
	16	${\underline{I}}_{PCC}^{-}$	[A]	$0^{\underline{/0}}$	$0.099^{\underline{/136.67{}^{\circ}}}$	$0.338^{\underline{/141.41{}^{\circ}}}$	$0.293^{\underline{/138.43{}^{\circ}}}$	$0.254^{\underline{/135{}^{\circ}}}$	$0.392^{\underline{/126.46{}^{\circ}}}$	$0.59^{\underline{/146.31{}^{\circ}}}$
	17	$k_{uI}^{-PCC}$	–	0	0.058	0.198	0.172	0.149	0.23	0.346
	18	${\underline{I}}_{PCC}^{0}$	[A]	$0^{\underline{/0}}$	$0.042^{\underline{/62.77{}^{\circ}}}$	$0.141^{\underline{/50.9{}^{\circ}}}$	$0.078^{\underline{/78.43{}^{\circ}}}$	$0.068^{\underline{/135{}^{\circ}}}$	$0.167^{\underline{/140.36{}^{\circ}}}$	$0.042^{\underline{/146.31{}^{\circ}}}$
	19	$k_{uI}^{0PCC}$	–	0	0.025	0.083	0.046	0.04	0.098	0.025

**Table 2.1:** Input data for numerical analysis of unbalanced capacitive compensation for the second group of particular load cases - Section 1 - Unbalanced load.

Comp.	Regime									
	Quantities			BCC 1_8	BCC 1_9	BCC 1_10	BCC 1_11	BCC 1_12	BCC 1_13	BCC 1_14
Load	1	$P_{A}^{load}$	[W]	200	250	200	250	200	250	300
	2	$P_{B}^{load}$	[W]	600	600	600	600	600	500	500
	3	$P_{C}^{load}$	[W]	400	350	400	350	400	450	400
	4	$P_{tot}^{load}$	[W]	1200	1200	1200	1200	1200	1200	1200
	5	$P_{av}^{load}$	[W]	400	400	400	400	400	400	400
	6	$R_{A}^{load}$	[Ω]	276.125	220.9	276.125	220.9	276.125	220.9	184.083333
	7	$R_{B}^{load}$	[Ω]	92.041667	92.041667	92.041667	92.041667	92.041667	110.45	110.45
	8	$R_{C}^{load}$	[Ω]	138.0625	157.785714	138.0625	157.785714	138.0625	122.722222	138.0625
	9	$Q_{A}^{load}$	[var]	400	400	500	500	600	600	600
	10	$Q_{B}^{load}$	[var]	700	700	800	800	800	800	800
	11	$Q_{C}^{load}$	[var]	1300	1300	1100	1100	1000	1000	1000
	12	$Q_{tot}^{load}$	[var]	2400	2400	2400	2400	2400	2400	2400
	13	$Q_{av}^{load}$	[var]	800	800	800	800	800	800	800
	14	$L_{A}^{load}$	[H]	0.439467	0.439467	0.351573	0.351573	0.292978	0.292978	0.292978
	15	$L_{B}^{load}$	[H]	0.251124	0.251124	0.219733	0.219733	0.219733	0.219733	0.219733
	16	$L_{C}^{load}$	[H]	0.13522	0.13522	0.159806	0.159806	0.175787	0.175787	0.175787
	17	$cos\varphi^{load}$	–	0.447214	0.447214	0.447214	0.447214	0.447214	0.447214	0.447214
	18	${\underline{I}}_{A}^{load}$	[A]	$1.903^{\underline{/-63.43{}^{\circ}}}$	$2.007^{\underline{/-57.99{}^{\circ}}}$	$2.292^{\underline{/-68.2{}^{\circ}}}$	$2.379^{\underline{/-63.43{}^{\circ}}}$	$2.691^{\underline{/-71.57{}^{\circ}}}$	$2.766^{\underline{/-67.38{}^{\circ}}}$	$2.855^{\underline{/-63.43{}^{\circ}}}$
	19	${\underline{I}}_{B}^{load}$	[A]	$3.923^{\underline{/-169.4{}^{\circ}}}$	$3.923^{\underline{/-169.4{}^{\circ}}}$	$4.255^{\underline{/-173.13{}^{\circ}}}$	$4.256^{\underline{/-173.13{}^{\circ}}}$	$4.255^{\underline{/-173.13{}^{\circ}}}$	$4.014^{\underline{/-177.99{}^{\circ}}}$	$4.014^{\underline{/-177.99{}^{\circ}}}$
	20	${\underline{I}}_{C}^{load}$	[A]	$5.788^{\underline{/47.1{}^{\circ}}}$	$5.729^{\underline{/45.07{}^{\circ}}}$	$4.981^{\underline{/49.98{}^{\circ}}}$	$4.912^{\underline{/47.65{}^{\circ}}}$	$4.583^{\underline{/51.8{}^{\circ}}}$	$4.666^{\underline{/54.23{}^{\circ}}}$	$4.583^{\underline{/51.8{}^{\circ}}}$
	21	${\underline{I}}_{load}^{+}$	[A]	$3.806^{\underline{/-63.43{}^{\circ}}}$	$3.806^{\underline{/-63.43{}^{\circ}}}$	$3.806^{\underline{/-63.43{}^{\circ}}}$	$3.806^{\underline{/-63.43{}^{\circ}}}$	$3.806^{\underline{/-63.43{}^{\circ}}}$	$3.806^{\underline{/-63.43{}^{\circ}}}$	$3.806^{\underline{/-63.43{}^{\circ}}}$
	22	${\underline{I}}_{load}^{-}$	[A]	$1.598^{\underline{/136.67{}^{\circ}}}$	$1.567^{\underline{/132.36{}^{\circ}}}$	$1.188^{\underline{/131.93{}^{\circ}}}$	$1.169^{\underline{/126.03{}^{\circ}}}$	$0.949^{\underline{/135{}^{\circ}}}$	$0.746^{\underline{/139.23{}^{\circ}}}$	$0.715^{\underline{/129.9{}^{\circ}}}$
	23	$k_{uI}^{-load}$	–	0.419923	0.411828	0.312199	0.307153	0.249401	0.195939	0.187795
	24	${\underline{I}}_{load}^{0}$	[A]	$0.681^{\underline{/62.77{}^{\circ}}}$	$0.686^{\underline{/52.47{}^{\circ}}}$	$0.397^{\underline{/98.26{}^{\circ}}}$	$0.335^{\underline{/81.52{}^{\circ}}}$	$0.254^{\underline{/135{}^{\circ}}}$	$0.371^{\underline{/101.41{}^{\circ}}}$	$0.304^{\underline{/83.79{}^{\circ}}}$
	25	$k_{uI}^{0load}$	–	0.17888	0.180226	0.104237	0.08798	0.066827	0.097594	0.079997
Weighting factors	26	$k_{Q}^{+}$	–	1	0.835658	0.818524	0.703317	0.690128	0.585686	0.4755
	27	$k_{Y}^{+}$	–	0.381198	0.323801	0.233663	0.195567	0.106763	0.217982	0.18126
	28	$k_{\Delta }^{+}$	–	0.618802	0.511857	0.584861	0.50775	0.583365	0.367704	0.294241
	29	$k^{-}$	–	1	1	1	1	1	1	1

**Table 2.2:** Output data for numerical analysis of unbalanced capacitive compensation for the second group of particular load cases - Section 2 - Yn compensator.

Comp.	Regime									
	Quantities			BCC 1_8	BCC 1_9	BCC 1_10	BCC 1_11	BCC 1_12	BCC 1_13	BCC 1_14
Compensator Yn	1	$C_{A}^{Y}$	[μF]	1.177463	0.194723	0.138366	0.045619	0.050755	0.187513	0.158093
	2	$C_{B}^{Y}$	[μF]	18.469082	12.494682	17.429985	12.345577	11.578501	16.70692	11.685839
	3	$C_{C}^{Y}$	[μF]	33.085678	32.102938	14.754962	14.662215	3.139605	13.259685	13.230264
	4	$P_{A}^{Y}$	[W]	0	0	0	0	0	0	0
	5	$P_{B}^{Y}$	[W]	0	0	0	0	0	0	0
	6	$P_{C}^{Y}$	[W]	0	0	0	0	0	0	0
	7	$Q_{A}^{Y}$	[var]	−20.428331	−3.378338	−2.400571	−0.791459	−0.880571	−3.253253	−2.742828
	8	$Q_{B}^{Y}$	[var]	−320.42833	−216.775798	−302.40057	−214.18891	−200.88057	−289.85579	−202.74282
	9	$Q_{C}^{Y}$	[var]	−574.01817	−556.968177	−255.99041	−254.38129	−54.47041	−230.04817	−229.53774
	10	$Q_{tot}^{Y}$	[var]	−914.87483	−777.122313	−560.79155	−469.36167	−256.23155	−523.15722	−435.02340
	11	$Q_{av}^{Y}$	[var]	−304.958	−259.041	−186.931	−156.454	−85.4105	−174.386	−145.008
	12	${\underline{I}}_{A}^{Y}$	[A]	$0.087^{\underline{/90{}^{\circ}}}$	$0.014^{\underline{/90{}^{\circ}}}$	$0.010^{\underline{/90{}^{\circ}}}$	$0.003^{\underline{/90{}^{\circ}}}$	$0.004^{\underline{/90{}^{\circ}}}$	$0.014^{\underline{/90{}^{\circ}}}$	$0.012^{\underline{/90{}^{\circ}}}$
	13	${\underline{I}}_{B}^{Y}$	[A]	$1.364^{\underline{/-30{}^{\circ}}}$	$0.922^{\underline{/-30{}^{\circ}}}$	$1.287^{\underline{/-30{}^{\circ}}}$	$0.911^{\underline{/-30{}^{\circ}}}$	$0.855^{\underline{/-30{}^{\circ}}}$	$1.233^{\underline{/-30{}^{\circ}}}$	$0.863^{\underline{/-30{}^{\circ}}}$
	14	${\underline{I}}_{C}^{Y}$	[A]	$2.443^{\underline{/-150{}^{\circ}}}$	$2.370^{\underline{/-150{}^{\circ}}}$	$1.089^{\underline{/-150{}^{\circ}}}$	$1.082^{\underline{/-150{}^{\circ}}}$	$0.232^{\underline{/-150{}^{\circ}}}$	$0.979^{\underline{/-150{}^{\circ}}}$	$0.977^{\underline{/-150{}^{\circ}}}$
	15	${\underline{I}}_{Y}^{+}$	[A]	$1.298^{\underline{/90{}^{\circ}}}$	$1.102^{\underline{/90{}^{\circ}}}$	$0.795^{\underline{/90{}^{\circ}}}$	$0.666^{\underline{/90{}^{\circ}}}$	$0.363^{\underline{/90{}^{\circ}}}$	$0.742^{\underline{/90{}^{\circ}}}$	$0.617^{\underline{/90{}^{\circ}}}$
	16	${\underline{I}}_{Y}^{-}$	[A]	$0.681^{\underline{/-62.77{}^{\circ}}}$	$0.686^{\underline{/-52.47{}^{\circ}}}$	$0.397^{\underline{/-98.26{}^{\circ}}}$	$0.335^{\underline{/-81.52{}^{\circ}}}$	$0.254^{\underline{/-135{}^{\circ}}}$	$0.371^{\underline{/-101.41{}^{\circ}}}$	$0.304^{\underline{/-83.79{}^{\circ}}}$
	17	${\underline{I}}_{Y}^{0}$	[A]	$0.681^{\underline{/-117.23{}^{\circ}}}$	$0.686^{\underline{/-127.53{}^{\circ}}}$	$0.397^{\underline{/-81.74{}^{\circ}}}$	$0.335^{\underline{/-98.48{}^{\circ}}}$	$0.254^{\underline{/-45{}^{\circ}}}$	$0.371^{\underline{/-78.59{}^{\circ}}}$	$0.304^{\underline{/-96.21{}^{\circ}}}$

**Table 2.3:** Output data for numerical analysis of unbalanced capacitive compensation for the second group of particular load cases - Section 3 - Δ compensator.

Comp.	Regime									
	Quantities			BCC 1_8	BCC 1_9	BCC 1_10	BCC 1_11	BCC 1_12	BCC 1_13	BCC 1_14
Compensator Δ	1	$C_{AB}^{\Delta }$	[μF]	0.637129	0.102605	0.115444	0.03948	0.092444	0.105436	0.085545
	2	$C_{BC}^{\Delta }$	[μF]	13.948224	13.413699	13.426538	13.350574	13.403538	6.760983	6.741092
	3	$C_{CA}^{\Delta }$	[μF]	13.948224	10.085925	13.426538	10.022801	13.403538	10.088757	6.741092
	4	$P_{A}^{\Delta }$	[W]	200	150	200	150	200	150	100
	5	$P_{B}^{\Delta }$	[W]	−200	−200	−200	−200	−200	−100	−100
	6	$P_{C}^{\Delta }$	[W]	0	50	0	50	0	−50	0
	7	$P_{tot}^{\Delta }$	[W]	0	0	0	0	0	0	0
	8	$P_{av}^{\Delta }$	[W]	0	0	0	0	0	0	0
	9	$Q_{A}^{\Delta }$	[var]	−379.57166	−265.148019	−352.41881	−261.86247	−351.22169	−265.29537	−177.65753
	10	$Q_{B}^{\Delta }$	[var]	−379.57166	−351.75056	−352.41881	−348.46502	−351.22169	−178.69283	−177.65753
	11	$Q_{C}^{\Delta }$	[var]	−725.98183	−611.558181	−698.82897	−608.27264	−697.63185	−438.50045	−350.86262
	12	$Q_{tot}^{\Delta }$	[var]	−1485.1251	−1228.45676	−1403.6666	−1218.6001	−1400.0752	−882.48867	−706.17769
	13	$Q_{av}^{\Delta }$	[var]	−495.042	−409.486	−467.889	−406.2	−466.692	−294.163	−235.393
	14	${\underline{I}}_{A}^{\Delta }$	[A]	$1.826^{\underline{/62.21{}^{\circ}}}$	$1.296^{\underline{/60.50{}^{\circ}}}$	$1.724^{\underline{/60.42{}^{\circ}}}$	$1.284^{\underline{/60.2{}^{\circ}}}$	$1.72^{\underline{/60.34{}^{\circ}}}$	$1.297^{\underline{/60.52{}^{\circ}}}$	$0.868^{\underline{/60.63{}^{\circ}}}$
	15	${\underline{I}}_{B}^{\Delta }$	[A]	$1.826^{\underline{/-2.21{}^{\circ}}}$	$1.722^{\underline{/-0.38{}^{\circ}}}$	$1.724^{\underline{/-0.42{}^{\circ}}}$	$1.71^{\underline{/-0.15{}^{\circ}}}$	$1.72^{\underline{/-0.34{}^{\circ}}}$	$0.871^{\underline{/-0.77{}^{\circ}}}$	$0.868^{\underline{/-0.63{}^{\circ}}}$
	16	${\underline{I}}_{C}^{\Delta }$	[A]	$3.089^{\underline{/-150{}^{\circ}}}$	$2.611^{\underline{/-154.67{}^{\circ}}}$	$2.974^{\underline{/-150{}^{\circ}}}$	$2.597^{\underline{/-154.7{}^{\circ}}}$	$2.969^{\underline{/-150{}^{\circ}}}$	$1.878^{\underline{/-143.49{}^{\circ}}}$	$1.493^{\underline{/-150{}^{\circ}}}$
	17	${\underline{I}}_{\Delta }^{+}$	[A]	$2.107^{\underline{/90{}^{\circ}}}$	$1.742^{\underline{/90{}^{\circ}}}$	$1.991^{\underline{/90{}^{\circ}}}$	$1.729^{\underline{/90{}^{\circ}}}$	$1.986^{\underline{/90{}^{\circ}}}$	$1.252^{\underline{/90{}^{\circ}}}$	$1.002^{\underline{/90{}^{\circ}}}$
	18	${\underline{I}}_{\Delta }^{-}$	[A]	$0.983^{\underline{/-30{}^{\circ}}}$	$0.886^{\underline{/-43.9{}^{\circ}}}$	$0.983^{\underline{/-30{}^{\circ}}}$	$0.886^{\underline{/-43.9{}^{\circ}}}$	$0.983^{\underline{/-30{}^{\circ}}}$	$0.65^{\underline{/-10.89{}^{\circ}}}$	$0.491^{\underline{/-30{}^{\circ}}}$
	19	${\underline{I}}_{\Delta }^{0}$	[A]	$0^{\underline{/0{}^{\circ}}}$	$0^{\underline{/0{}^{\circ}}}$	$0^{\underline{/0{}^{\circ}}}$	$0^{\underline{/0{}^{\circ}}}$	$0^{\underline{/0{}^{\circ}}}$	$0^{\underline{/0{}^{\circ}}}$	$0^{\underline{/0{}^{\circ}}}$

**Table 2.4:** Output data for numerical analysis of unbalanced capacitive compensation for the second group of particular load cases - Section 4 - Yn+Δ compensator.

Comp.	Regime									
	Quantities			BCC 1_8	BCC 1_9	BCC 1_10	BCC 1_11	BCC 1_12	BCC 1_13	BCC 1_14
Compensator Yn + Δ	1	$P_{A}^{comp}$	[W]	200	150	200	150	200	150	100
	2	$P_{B}^{comp}$	[W]	−200	−200	−200	−200	−200	−100	−100
	3	$P_{C}^{comp}$	[W]	0	50	0	150	0	−50	0
	4	$P_{tot}^{comp}$	[W]	0	0	0	0	0	0	0
	5	$P_{av}^{comp}$	[W]	0	0	0	0	0	0	0
	6	$Q_{A}^{comp}$	[var]	−400	−268.526	−354.819	−262.654	−352.102	−268.549	−180.4
	7	$Q_{B}^{comp}$	[var]	−700	−568.526	−654.819	−562.654	−552.102	−468.549	−380.4
	8	$Q_{C}^{comp}$	[var]	−1300	−1168.526	−954.819	−862.654	−752.102	−668.549	−580.4
	9	$Q_{tot}^{comp}$	[var]	−2400	−2005.578	−1964.458	−1687.962	−1656.307	−1405.646	−1141.201
	10	$Q_{av}^{comp}$	[var]	−800	−668.526	−654.819	−562.654	−552.102	−468.549	−380.4
	11	${\underline{I}}_{A}^{comp}$	[A]	$1.903^{\underline{/63.43{}^{\circ}}}$	$1.309^{\underline{/60.81{}^{\circ}}}$	$1.733^{\underline{/60.59{}^{\circ}}}$	$1.287^{\underline{/60.27{}^{\circ}}}$	$1.723^{\underline{/60.4{}^{\circ}}}$	$1.309^{\underline{/60.81{}^{\circ}}}$	$0.878^{\underline{/61{}^{\circ}}}$
	12	${\underline{I}}_{B}^{comp}$	[A]	$3.098^{\underline{/-14.05{}^{\circ}}}$	$2.565^{\underline{/-10.62{}^{\circ}}}$	$2.914^{\underline{/-13.02{}^{\circ}}}$	$2.541^{\underline{/-10.43{}^{\circ}}}$	$2.499^{\underline{/-10.09{}^{\circ}}}$	$2.039^{\underline{/-17.95{}^{\circ}}}$	$1.674^{\underline{/-15.27{}^{\circ}}}$
	13	${\underline{I}}_{C}^{comp}$	[A]	$5.532^{\underline{/-150{}^{\circ}}}$	$4.977^{\underline{/-152.45{}^{\circ}}}$	$4.063^{\underline{/-150{}^{\circ}}}$	$3.677^{\underline{/-153.32{}^{\circ}}}$	$3.2^{\underline{/-150{}^{\circ}}}$	$2.853^{\underline{/-145.72{}^{\circ}}}$	$2.47^{\underline{/-150{}^{\circ}}}$
	14	${\underline{I}}_{comp}^{+}$	[A]	$3.404^{\underline{/90{}^{\circ}}}$	$2.845^{\underline{/90{}^{\circ}}}$	$2.786^{\underline{/90{}^{\circ}}}$	$2.394^{\underline{/90{}^{\circ}}}$	$2.349^{\underline{/90{}^{\circ}}}$	$1.994^{\underline{/90{}^{\circ}}}$	$1.619^{\underline{/90{}^{\circ}}}$
	15	${\underline{I}}_{comp}^{-}$	[A]	$1.598^{\underline{/-43.33{}^{\circ}}}$	$1.567^{\underline{/-47.64{}^{\circ}}}$	$1.188^{\underline{/-48.07{}^{\circ}}}$	$1.169^{\underline{/-53.97{}^{\circ}}}$	$0.949^{\underline{-45{}^{\circ}}}$	$0.746^{\underline{/-40.77{}^{\circ}}}$	$0.715^{\underline{/-50.1{}^{\circ}}}$
	16	${\underline{I}}_{comp}^{0}$	[A]	$0.681^{\underline{/-117.23{}^{\circ}}}$	$0.686^{\underline{/-127.53{}^{\circ}}}$	$0.397^{\underline{/-81.74{}^{\circ}}}$	$0.335^{\underline{/-98.48{}^{\circ}}}$	$0.254^{\underline{/-45{}^{\circ}}}$	$0.371^{\underline{/-78.59{}^{\circ}}}$	$0.304^{\underline{/-96.21{}^{\circ}}}$

**Table 2.5:** Output data for numerical analysis of unbalanced capacitive compensation for the second group of particular load cases - Section 2 - PCC.

Comp.	Regime									
	Quantities			BCC 1_8	BCC 1_9	BCC 1_10	BCC 1_11	BCC 1_12	BCC 1_13	BCC 1_14
PCC	1	$P_{A}^{PCC}$	[W]	400	400	400	400	400	400	400
	2	$P_{B}^{PCC}$	[W]	400	400	400	400	400	400	400
	3	$P_{C}^{PCC}$	[W]	400	400	400	400	400	400	400
	4	$P_{tot}^{PCC}$	[W]	1200	1200	1200	1200	1200	1200	1200
	5	$P_{av}^{PCC}$	[W]	400	400	400	400	400	400	400
	6	$Q_{A}^{PCC}$	[var]	0	131.473642	145.180614	237.346062	247.897735	331.451369	419.599633
	7	$Q_{B}^{PCC}$	[var]	0	131.473642	145.180614	237.346062	247.897735	331.451369	419.599633
	8	$Q_{C}^{PCC}$	[var]	0	131.473642	145.180614	237.346062	247.897735	331.451369	419.599633
	9	$Q_{tot}^{PCC}$	[var]	0	394.4209	435.5418	712.0382	743.6932	994.3541	1258.799
	10	$Q_{av}^{PCC}$	[var]	0	131.473642	145.180614	237.346062	247.897735	331.451369	419.599633
	11	$cos\varphi^{PCC}$	–	1	0.95	0.94	0.86	0.85	0.77	0.69
	12	${\underline{I}}_{A}^{PCC}$	[A]	$1.702^{\underline{/0{}^{\circ}}}$	$1.792^{\underline{/-18.19{}^{\circ}}}$	$1.811^{\underline{/-19.95{}^{\circ}}}$	$1.979^{\underline{/-30.68{}^{\circ}}}$	$2.003^{\underline{/-31.79{}^{\circ}}}$	$2.211^{\underline{/-39.65{}^{\circ}}}$	$2.467^{\underline{/-46.37{}^{\circ}}}$
	13	${\underline{I}}_{B}^{PCC}$	[A]	$1.702^{\underline{/-120{}^{\circ}}}$	$1.792^{\underline{/-138.19{}^{\circ}}}$	$1.811^{\underline{/-139.95{}^{\circ}}}$	$1.979^{\underline{/-150.68{}^{\circ}}}$	$2.003^{\underline{/-151.79{}^{\circ}}}$	$2.211^{\underline{/-159.65{}^{\circ}}}$	$2.467^{\underline{/-166.37{}^{\circ}}}$
	14	${\underline{I}}_{C}^{PCC}$	[A]	$1.702^{\underline{/120{}^{\circ}}}$	$1.792^{\underline{/101.81{}^{\circ}}}$	$1.811^{\underline{/100.05{}^{\circ}}}$	$1.979^{\underline{/89.32{}^{\circ}}}$	$2.003^{\underline{/88.21{}^{\circ}}}$	$2.211^{\underline{/80.35{}^{\circ}}}$	$2.467^{\underline{/73.63{}^{\circ}}}$
	15	${\underline{I}}_{PCC}^{+}$	[A]	$1.702^{\underline{/0{}^{\circ}}}$	$1.792^{\underline{/-18.19{}^{\circ}}}$	$1.811^{\underline{/-19.95{}^{\circ}}}$	$1.979^{\underline{/-30.68{}^{\circ}}}$	$2.003^{\underline{/-31.79{}^{\circ}}}$	$2.211^{\underline{/-39.65{}^{\circ}}}$	$2.467^{\underline{/-46.37{}^{\circ}}}$
	16	${\underline{I}}_{PCC}^{-}$	[A]	$0^{\underline{/0{}^{\circ}}}$	$0^{\underline{/0{}^{\circ}}}$	$0^{\underline{/0{}^{\circ}}}$	$0^{\underline{/0{}^{\circ}}}$	$0^{\underline{/0{}^{\circ}}}$	$0^{\underline{/0{}^{\circ}}}$	$0^{\underline{/0{}^{\circ}}}$
	17	$k_{uI}^{-PCC}$	–	0	0	0	0	0	0	0
	18	${\underline{I}}_{PCC}^{0}$	[A]	$0^{\underline{/0{}^{\circ}}}$	$0^{\underline{/0{}^{\circ}}}$	$0^{\underline{/0{}^{\circ}}}$	$0^{\underline{/0{}^{\circ}}}$	$0^{\underline{/0{}^{\circ}}}$	$0^{\underline{/0{}^{\circ}}}$	$0^{\underline{/0{}^{\circ}}}$
	19	$k_{uI}^{0PCC}$	–	0	0	0	0	0	0	0

Cases 1 and 8 of the load structure are the same. In fact, 13 particular cases of load were presented.

**Fig. 1:**
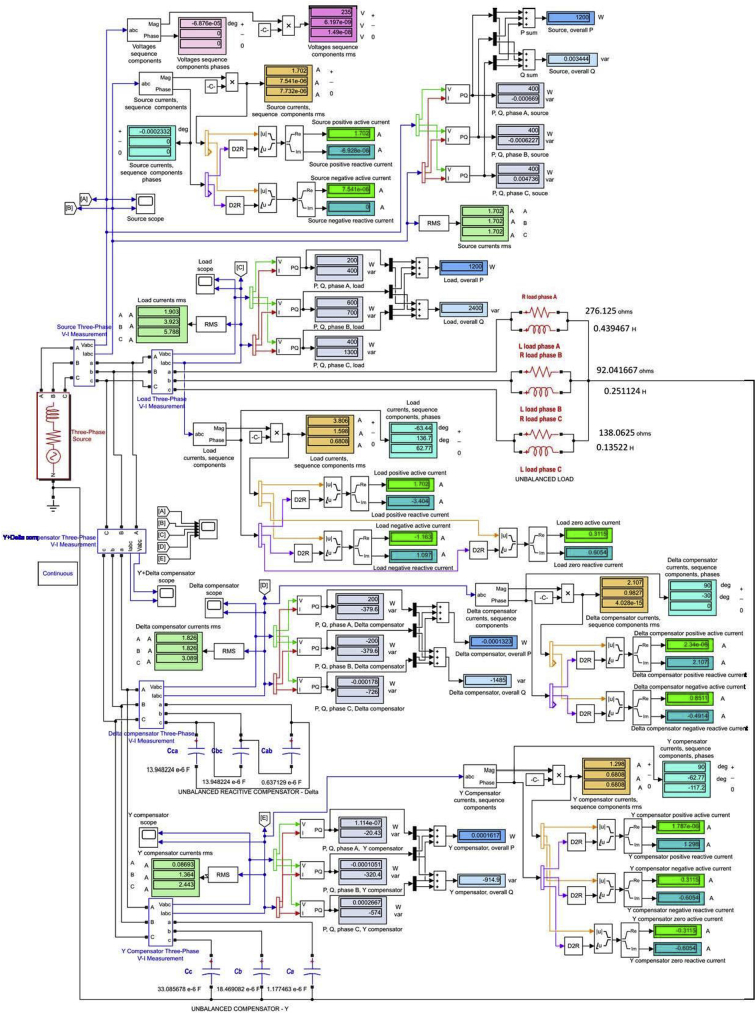
The model and simulation data of unbalanced capacitive compensation for the particular load case to which BCC 1_1(8) was installed.

**Fig. 2:**
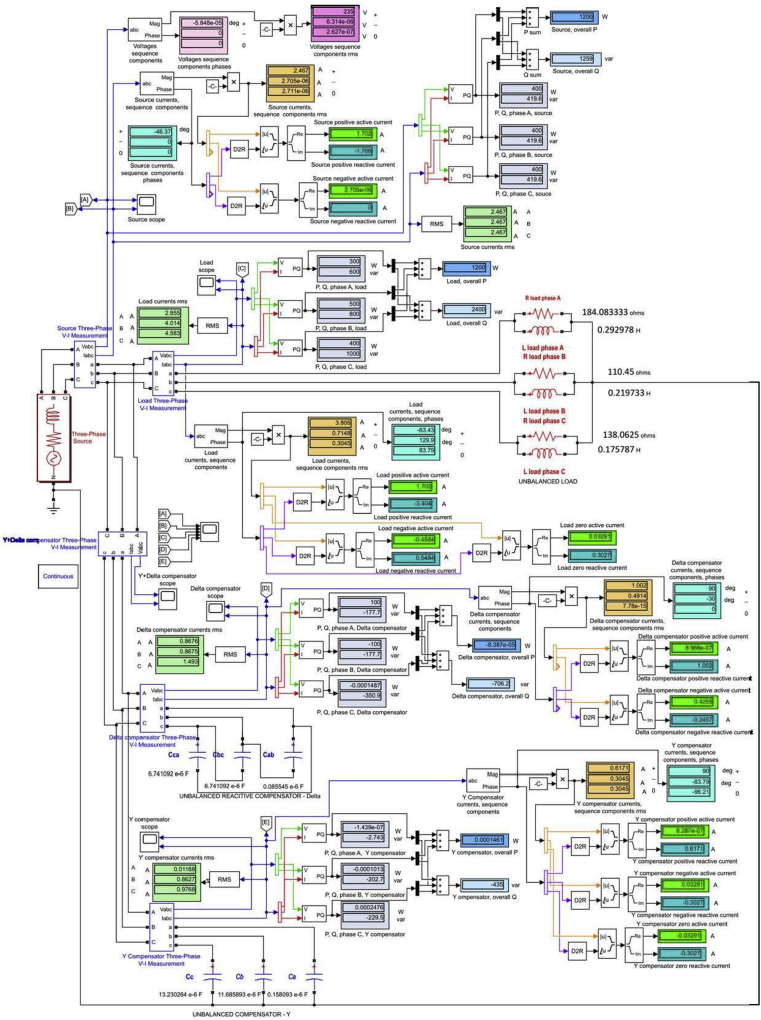
The model and simulation data of unbalanced capacitive compensation for the particular load case to which BCC 1_14 was installed.

**Fig. 3:**
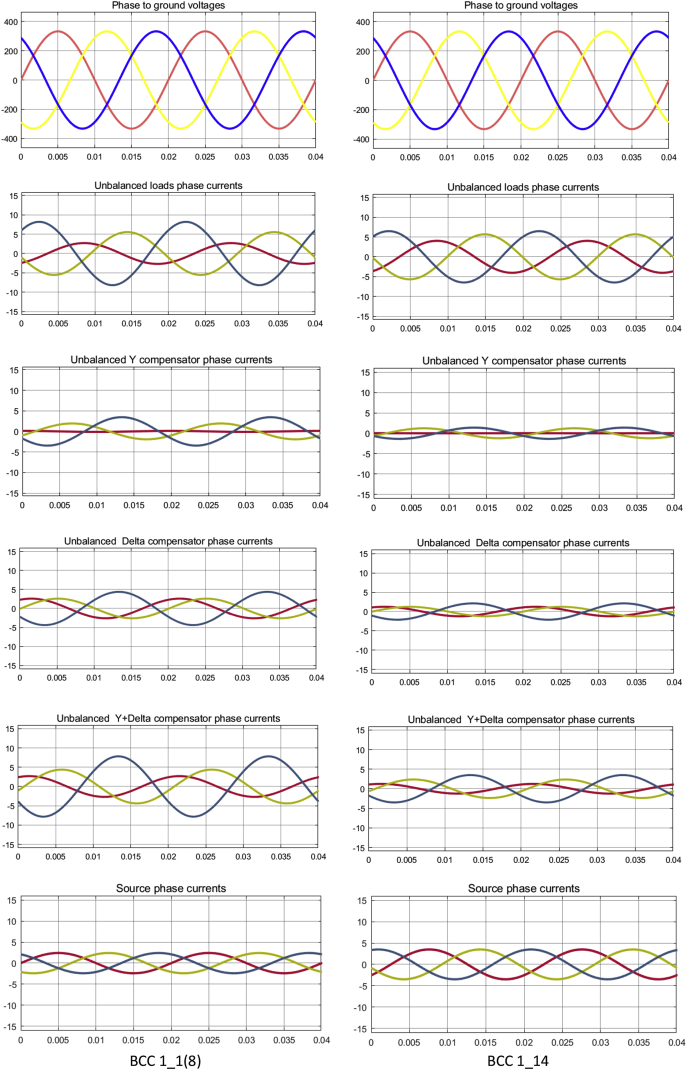
The phase voltages and currents waveforms of the three-phase circuit, in the five analyzed sections, for the particular load cases where a BCC was installed: 1_1 (8) and 1_14 respectively (Matlab-Simulink).

The output data of the numerical analysis are practically identical to those obtained by modeling and simulation performed using the Matlab-Simulink. To demonstrate this statement, the models and the simulation data for only two of the particular cases are presented: the case of the load where BCC1_1 (identical to BCC1_8) was installed (Fig. 1) and the case of the load where BCC1_14 was installed (Fig. 2). In Fig. 3 are presented, face to face, the phase voltages and currents waveforms in the five analyzed sections, for the two particular cases modeled and simulated by Matlab-Simulink.

## 2 Experimental design, materials and methods

In order to demonstrate the correctness of the method of determination of the unbalanced capacitive compensator susceptances values respectively of the values obtained by power flow calculation (Mathcad) and modeling-simulation (Matlab-Simulink), experimental laboratory determinations were performed. For this purpose, only the particular case of BCC 5 use, defined in [1], which is more interesting than the others, because it allows totally load balancing, was considered.

**Fig. 4:**
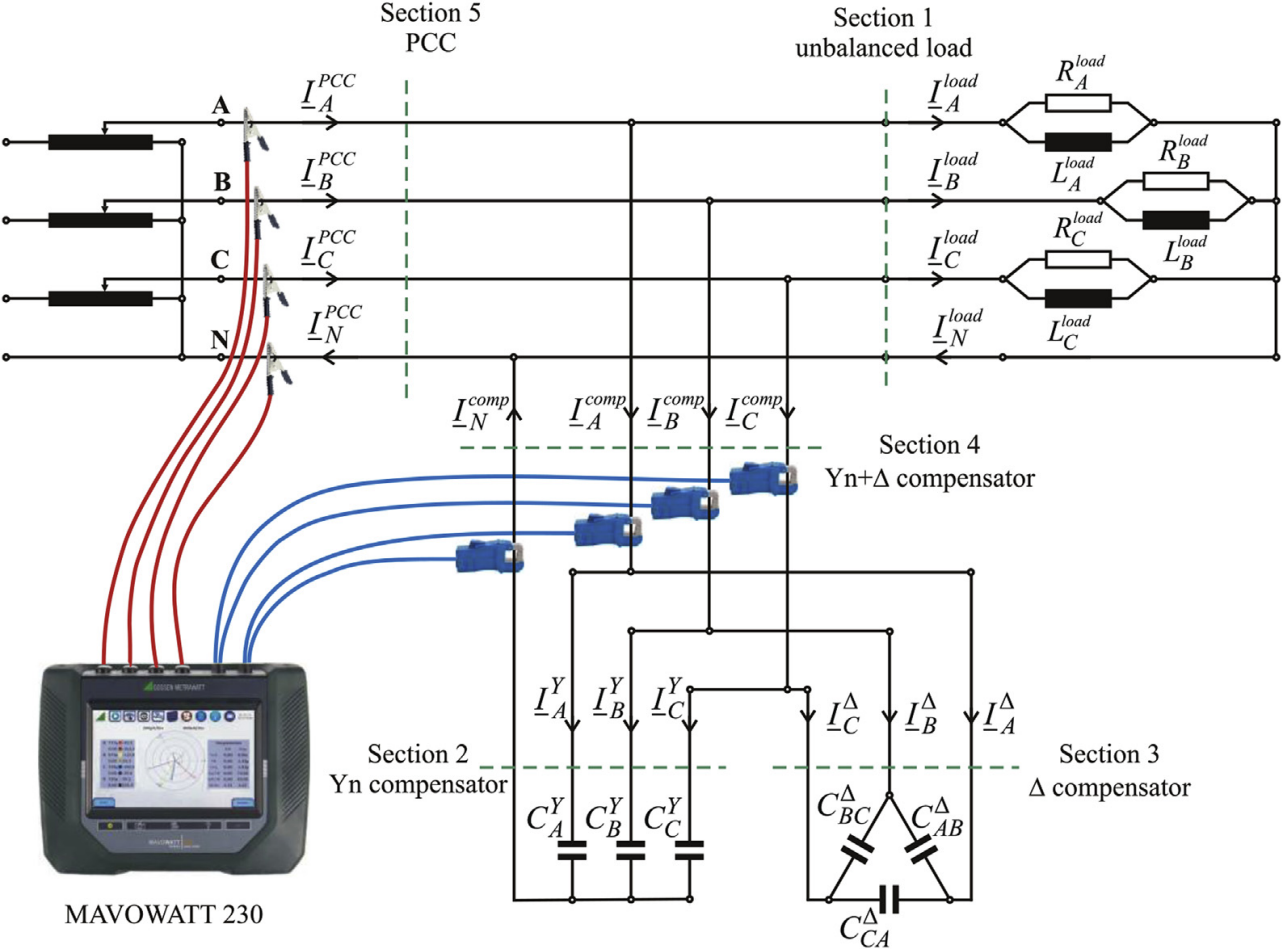
Circuit schema used in experimental determinations.

The three-phase circuits were built based on the simplified electrical schema presented in [1]. The installation of the circuit elements and the recording equipment is shown in Fig. 4. The circuit elements have been adjusted so that the values of the equivalent parameters are as close as possible to those applied in the numerical study of normal operation regime for both unbalanced load and for BCC 5.

A power quality analyzer, type MAVOWATT 230, was used for measurements.

Fig. 4 presents the electrical schema of the circuits built in the laboratory to perform experimental determinations.

For the five sections of interest of the three-phase circuit, the following data were extracted in graphical form and they were included in [1]:

• the rms values of phase voltages and currents,
• the values of active and reactive powers;
• the waveforms of the phase voltages and corresponding currents;
• the phasor diagrams of phase voltages and currents.

As expected, the data provided by experimental determinations are not identical to those provided by calculation (Mathcad) or simulation (Matlab-Simulink). In fact, simulation is based on numerical methods and algorithms for solving electrical circuits, so it is also a calculation. As it can be seen, between the data provided by the calculation and simulation, there is an almost perfect identity.

Experimental determinations were performed in conditions more or less close to ideal ones, in which the calculation and simulation were done as follows:

The power supply of the circuit was a three-phase autotransformer connected directly to the laboratory installation with a rated voltage of 230/400 V. Thus, due to both the supply network and the constructive unbalance of the autotransformer, the three-phase voltage set is neither symmetrical either as rms values and as phase shift. These non-symmetries are, however, very small. The percentage deviations from the reference values (values considered in numerical analysis and simulation) have the maximum values of 2.52% for rms values and 0.33% for phase shifts. The fact that the voltage asymmetries provided by the source used for experimental determinations can be neglected is also supported by the very small values of their negative and zero sequence components, which do not exceed the value of 0.6% relative to the positive sequence component.

The second cause of deviations of the values obtained by experimental determinations from those obtained by calculation, is the presence of voltages and currents harmonic regime. The voltage distortion mainly due to the network and the non-linearity of the autotransformer. However, the total harmonic distortion of the phase voltages does not exceed 4%. The currents distortion come from the distorted supply voltages, but at the same time it is amplified due to the presence of non-linear circuit elements: single-phase ferromagnetic core coils used as inductive loads, respectively single-phase electrolytic capacitors used in the construction of the two three-phase compensators. However, the THD values for currents remained within reasonable limits, which did not exceed 15%.

The third cause of the deviations comes from the fact that the coils and capacitors intervene in the circuit, in addition to the reactive, dominant components, by their active components corresponding to the losses of active power produced in the windings, the ferromagnetic cores and the dielectric materials.

The fourth cause of deviations is itself the instrument of measurement. The Mavowatt 230 is actually a digital power quality analyzer. The measured voltages were acquired in a single node (at source) but in order to obtain the currents respective the powers in the five sections of the circuit, the measurements were repeated after the current clamps were moved. The measurement errors are very small, but especially in the case of currents, they are influenced by the rms measured values. It is known that measuring currents by using current transformers (current clamps) at much lower rms values than rated currents, the measurement errors are very high (even over ±5%). When measuring phase shifts (angles), errors are even greater. In addition, measured voltages and currents distortion leads to an additional increase in measurement errors.

To facilitate a direct comparison of experimentally obtained values with those obtained from the calculation they were grouped face-to-face in Table 3.1, Table 3.2, Table 3.3, Table 3.4, Table 3.5. Also here it can be seen the percentages of deviations for all determined amounts.

**Table 3.1:** Comparison between the values obtained by numerical analysis (simulation) and the values obtained by experimental determinations – Section 1 - unbalanced load.

Component	Regime			BCC 5		
	Amounts			Ideal BCC (Mathcad, Simulink)	Real BCC (Experiment)	Deviation [%]
Unbalanced load	1	$P_{A}^{load}$	[W]	300	299.77	−0.74%
	2	$P_{B}^{load}$	[W]	500	490.39	−1.92%
	3	$P_{C}^{load}$	[W]	1000	991.32	−0.87%
	4	$P_{tot}^{load}$	[W]	1800	1779.50	−1.14%
	5	$P_{av}^{load}$	[W]	600	593.16	−1.14%
	6	$Q_{A}^{load}$	[var]	1000	1032.90	3.29%
	7	$Q_{B}^{load}$	[var]	200	221.57	10.79%
	8	$Q_{C}^{load}$	[var]	1500	1540.10	2.67%
	9	$Q_{tot}^{load}$	[var]	2700	2794.60	3.50%
	10	$Q_{av}^{load}$	[var]	900	931.53	3.50%
	11	$cos\varphi^{load}$	–	0.5547	0.537	−3.19%
	12	${\underline{I}}_{A}^{load}$	[A]	$4.539^{\underline{/-73.3{}^{\circ}}}$	$4.670^{\underline{/-73.6{}^{\circ}}}$	$2.89{\%}^{\underline{/0.41\%}}$
	13	${\underline{I}}_{B}^{load}$	[A]	$2.341^{\underline{/-141.8{}^{\circ}}}$	$2.34^{\underline{/-144.0{}^{\circ}}}$	$-0.04{\%}^{\underline{/-1.55\%}}$
	14	${\underline{I}}_{C}^{load}$	[A]	$7.838^{\underline{/63.7{}^{\circ}}}$	$7.88^{\underline{/62.7{}^{\circ}}}$	$0.54{\%}^{\underline{/-1.57\%}}$
	15	${\underline{I}}_{load}^{+}$	[A]	$4.703^{\underline{/-56.3{}^{\circ}}}$	4.78	1.64%
	16	${\underline{I}}_{load}^{-}$	[A]	$2.435^{\underline{/-159.7{}^{\circ}}}$	2.42	−0.62%
	17	$k_{uI}^{-load}$	–	0.518	0.507	−2.12%
	18	${\underline{I}}_{load}^{0}$	[A]	$1.062^{\underline{/22.7{}^{\circ}}}$	1.09	2.64%
	19	$k_{uI}^{0load}$	–	0.226	0.2273	0.58%

**Table 3.2:** Comparison between the values obtained by numerical analysis (simulation) and the values obtained by experimental determinations – Section 2 – Yn compensator.

Component	Regime			BCC 5		
	Amounts			Ideal BCC (Mathcad, Simulink)	Real BCC (Experiment)	Deviation [%]
Yn Compensator	1	$P_{A}^{Y}$	[W]	0	−0.173	–
	2	$P_{B}^{Y}$	[W]	0	0.512	–
	3	$P_{C}^{Y}$	[W]	0	−0.114	–
	4	$P_{tot}^{Y}$	[W]	0	0.225	–
	5	$P_{av}^{Y}$	[W]	0	0.075	–
	6	$Q_{A}^{Y}$	[var]	−116.26	−115.08	−1.01%
	7	$Q_{B}^{Y}$	[var]	−9.08	−9.24	1.76%
	8	$Q_{C}^{Y}$	[var]	−789.46	−797.71	1.05%
	9	$Q_{tot}^{Y}$	[var]	−914.80	−922.03	0.79%
	10	$Q_{av}^{Y}$	[var]	−304.93	−307.34	0.79%
	11	${\underline{I}}_{A}^{Y}$	[A]	$0.505^{\underline{/90{}^{\circ}}}$	$0.50^{\underline{/90.1{}^{\circ}}}$	$0.99{\%}^{\underline{/0.11\%}}$
	12	${\underline{I}}_{B}^{Y}$	[A]	$0.039^{\underline{/-30{}^{\circ}}}$	$0.04^{\underline{/-32.8{}^{\circ}}}$	$-2.56{\%}^{\underline{/9.33\%}}$
	13	${\underline{I}}_{C}^{Y}$	[A]	$3.432^{\underline{/-150{}^{\circ}}}$	$3.46^{\underline{/-150.1{}^{\circ}}}$	$0.82{\%}^{\underline{/0.07\%}}$
	14	${\underline{I}}_{Y}^{+}$	[A]	$1.326^{\underline{/90{}^{\circ}}}$	1.33	2.30%
	15	${\underline{I}}_{Y}^{-}$	[A]	$1.062^{\underline{/-22.7{}^{\circ}}}$	1.07	0.75%
	16	${\underline{I}}_{Y}^{0}$	[A]	$1.062^{\underline{/-157.3{}^{\circ}}}$	1.07	0.75%

**Table 3.3:** Comparison between the values obtained by numerical analysis (simulation) and the values obtained by experimental determinations – Section 3 – Δ compensator.

Component	Regime			BCC 5		
	Amounts			Ideal BCC (Mathcad, Simulink)	Real BCC (Experiment)	Deviation [%]
Δ Compensator	1	$P_{A}^{\Delta }$	[W]	300	297.79	−0.74%
	2	$P_{B}^{\Delta }$	[W]	100	111.35	11.35%
	3	$P_{C}^{\Delta }$	[W]	−400	−393.80	−1.55%
	4	$P_{tot}^{\Delta }$	[W]	0	15.34	–
	5	$P_{av}^{\Delta }$	[W]	0	5.11	–
	6	$Q_{A}^{\Delta }$	[var]	−883.74	−115.08	0.58%
	7	$Q_{B}^{\Delta }$	[var]	−190.92	−9.24	10.21%
	8	$Q_{C}^{\Delta }$	[var]	−710.54	−797.71	1.13%
	9	$Q_{tot}^{\Delta }$	[var]	−1785.20	−922.03	1.83%
	10	$Q_{av}^{\Delta }$	[var]	−595.07	−307.34	1.83%
	11	${\underline{I}}_{A}^{\Delta }$	[A]	$4.058^{\underline{/71.2{}^{\circ}}}$	$0.50^{\underline{/90.1{}^{\circ}}}$	$1.28{\%}^{\underline{/0.28\%}}$
	12	${\underline{I}}_{B}^{\Delta }$	[A]	$0.937^{\underline{/-57.6{}^{\circ}}}$	$0.04^{\underline{/-32.8{}^{\circ}}}$	$10.99{\%}^{\underline{/-0.17\%}}$
	13	${\underline{I}}_{C}^{\Delta }$	[A]	$3.545^{\underline{/-120.6{}^{\circ}}}$	$3.46^{\underline{/-150.1{}^{\circ}}}$	$0.42{\%}^{\underline{/0.50\%}}$
	14	${\underline{I}}_{\Delta }^{+}$	[A]	$2.587^{\underline{/90{}^{\circ}}}$	1.33	2.05%
	15	${\underline{I}}_{\Delta }^{-}$	[A]	$1.810^{\underline{/43.9{}^{\circ}}}$	1.07	−1.10%
	16	${\underline{I}}_{\Delta }^{0}$	[A]	$0^{\underline{/0{}^{\circ}}}$	1.07	–

**Table 3.4:** Comparison between the values obtained by numerical analysis (simulation) and the values obtained by experimental determinations – Section 4 – Yn+Δ compensator.

Component	Regime			BCC 5		
	Amounts			Ideal BCC (Mathcad, Simulink)	Real BCC (Experiment)	Deviation [%]
Yn + Δ Compensator	1	$P_{A}^{comp}$	[W]	300	302.23	0.74%
	2	$P_{B}^{comp}$	[W]	100	112.85	12.85%
	3	$P_{C}^{comp}$	[W]	−400	−399.50	−0.13%
	4	$P_{tot}^{comp}$	[W]	0	15.59	–
	5	$P_{av}^{comp}$	[W]	0	4.86	–
	6	$Q_{A}^{comp}$	[var]	−1000	−1019.0	−1.90%
	7	$Q_{B}^{comp}$	[var]	−200	−221.8	10.21%
	8	$Q_{C}^{comp}$	[var]	−1500	−1532.9	2.19%
	9	$Q_{tot}^{comp}$	[var]	−2700	−2773.8	2.73%
	10	$Q_{av}^{comp}$	[var]	−900	−924.6	2.73%
	11	${\underline{I}}_{A}^{comp}$	[A]	$4.539^{\underline{/73.3{}^{\circ}}}$	$4.63^{\underline{/90.1{}^{\circ}}}$	$2.00{\%}^{\underline{/0.14\%}}$
	12	${\underline{I}}_{B}^{comp}$	[A]	$0.972^{\underline{/-56.6{}^{\circ}}}$	$1.08^{\underline{/-32.8{}^{\circ}}}$	$11.11{\%}^{\underline{/0.00\%}}$
	13	${\underline{I}}_{C}^{comp}$	[A]	$6.750^{\underline{/-135.1{}^{\circ}}}$	$6.83^{\underline{/-150.1{}^{\circ}}}$	$1.19{\%}^{\underline{/0.37\%}}$
	14	${\underline{I}}_{comp}^{+}$	[A]	$3.913^{\underline{/90{}^{\circ}}}$	4.01	2.48%
	15	${\underline{I}}_{comp}^{-}$	[A]	$2.435^{\underline{/-20.3{}^{\circ}}}$	2.43	−0.21%
	16	${\underline{I}}_{comp}^{0}$	[A]	$1.062^{\underline{/-157.3{}^{\circ}}}$	1.07	0.75%

**Table 3.5:** Comparison between the values obtained by numerical analysis (simulation) and the values obtained by experimental determinations – Section 5 - PCC.

Component	Regime			BCC 5		
	Amounts			Ideal BCC (Mathcad, Simulink)	Real BCC (Experiment)	Deviation [%]
**PCC**	1	$P_{A}^{PCC}$	[W]	600	601.73	0.29%
	2	$P_{B}^{PCC}$	[W]	600	600.77	0.13%
	3	$P_{C}^{PCC}$	[W]	600	601.36	0.23%
	4	$P_{tot}^{PCC}$	[W]	1800	1803.39	0.19%
	5	$P_{av}^{PCC}$	[W]	600	601.13	0.19%
	6	$Q_{A}^{PCC}$	[var]	0	8.85	–
	7	$Q_{B}^{PCC}$	[var]	0	3.15	–
	8	$Q_{C}^{PCC}$	[var]	0	5.07	–
	9	$Q_{tot}^{PCC}$	[var]	0	17.07	–
	10	$Q_{av}^{PCC}$	[var]	0	5.69	–
	11	$cos\varphi^{+PCC}$	–	1	1	0.00%
	12	${\underline{I}}_{A}^{PCC}$	[A]	$2.609^{\underline{/0{}^{\circ}}}$	$2.64^{\underline{/-1.4{}^{\circ}}}$	$1.19{\%}^{\underline{/-}}$
	13	${\underline{I}}_{B}^{PCC}$	[A]	$2.609^{\underline{/-120{}^{\circ}}}$	$2.62^{\underline{/-120.1{}^{\circ}}}$	$0.42{\%}^{\underline{/0.08\%}}$
	14	${\underline{I}}_{C}^{PCC}$	[A]	$2.609^{\underline{/120{}^{\circ}}}$	$2.60^{\underline{/119.4{}^{\circ}}}$	$-0.34{\%}^{\underline{/-0.50\%}}$
	15	${\underline{I}}_{PCC}^{+}$	[A]	$2.609^{\underline{/0{}^{\circ}}}$	2.62	0.42%
	16	${\underline{I}}_{PCC}^{-}$	[A]	$0.0^{\underline{/0{}^{\circ}}}$	0.013	–
	17	$k_{uI}^{-PCC}$	–	0	0.005	–
	18	${\underline{I}}_{PCC}^{0}$	[A]	$0.0^{\underline{/0{}^{\circ}}}$	0.027	–
	19	$k_{uI}^{0PCC}$	–	0	0.0105	–
